# Lepidoptera demonstrate the relevance of Murray’s Law to circulatory systems with tidal flow

**DOI:** 10.1186/s12915-021-01130-0

**Published:** 2021-09-15

**Authors:** Sandra R. Schachat, C. Kevin Boyce, Jonathan L. Payne, David Lentink

**Affiliations:** 1 Department of Geological Sciences, Stanford University, Stanford, USA; 2 Department of Mechanical Engineering, Stanford University, Stanford, USA; 3 Faculty of Science and Engineering, University of Groningen, Groningen, The Netherlands

**Keywords:** Fluid transport, Hemolypmh, Insect, Tracheae, Venation

## Abstract

**Background:**

Murray’s Law, which describes the branching architecture of bifurcating tubes, predicts the morphology of vessels in many amniotes and plants. Here, we use insects to explore the universality of Murray’s Law and to evaluate its predictive power for the wing venation of Lepidoptera, one of the most diverse insect orders. Lepidoptera are particularly relevant to the universality of Murray’s Law because their wing veins have tidal, or oscillatory, flow of air and hemolymph. We examined over one thousand wings representing 667 species of Lepidoptera.

**Results:**

We found that veins with a diameter above approximately 50 microns conform to Murray’s Law, with veins below 50 microns in diameter becoming less and less likely to conform to Murray’s Law as they narrow. The minute veins that are most likely to deviate from Murray’s Law are also the most likely to have atrophied, which prevents efficient fluid transport regardless of branching architecture. However, the veins of many taxa continue to branch distally to the areas where they atrophied, and these too conform to Murray’s Law at larger diameters (e.g., Sesiidae).

**Conclusions:**

This finding suggests that conformity to Murray’s Law in larger taxa may reflect requirements for structural support as much as fluid transport, or may indicate that selective pressures for fluid transport are stronger during the pupal stage—during wing development prior to vein atrophy—than the adult stage. Our results increase the taxonomic scope of Murray’s Law and provide greater clarity about the relevance of body size.

**Supplementary Information:**

The online version contains supplementary material available at (10.1186/s12915-021-01130-0).

## Background

Biologists have long searched for universal “laws” that govern life on earth [1]. The wings of insects provide an amenable system for evaluating relevant biological laws because insects have the highest described species diversity of any group of animals [2] and their wings vary tremendously in terms of size, shape, and biomechanics [3]. Furthermore, insect wings serve a range of functions from thermoregulation to flight to sexual signaling, and the veins within insect wings serve sensory, structural, and circulatory roles [4].

The transport of air and hemolymph through the veins of insect wings is of particular relevance because the circulatory systems of animals and the vascular systems of plants present a rare opportunity to identify fundamental generalities that apply to more than one kingdom of multicellular life [5]. These efforts have expanded to include the rate of flow through vessels that bifurcate, such as an artery that branches into arterioles [6] or a vein in an insect wing. Flow through a bifurcating tube is often evaluated with the equation:

$ d_{0}^{k} = d_{1}^{k} + d_{2}^{k} +... + d_{n}^{k}$

wherein a single tube with diameter *d*_0_ branches into multiple tubes with diameters *d*_1_,*d*_2_,..., *d*_*n*_. Certain values of *k*, the “junction exponent” [7], represent distinct biophysical optima. When *k* = 2, the branching architecture conforms to “da Vinci’s Law” [8] and flow velocity is conserved across the point of bifurcation; when *k* = 3, the branching architecture conforms to “Murray’s Law” [9] and transport capacity is conserved across the point of bifurcation; when *k* = 4, resistance to flow is conserved across the point of bifurcation [10].

At the biophysical optimum described by Murray’s Law, a cost function is preserved before and after the vessel bifurcates [11]. There are two costs to moving fluid through a biological vessel. The first cost is the energy, or power, that fluid transport requires. This cost decreases as vessel radius (*r*) increases (power for steady flow ∼1/*r*^4^), assuming viscosity, kinetic, and gravity terms remain constant throughout the network. The second cost is the development and maintenance of the living cells that compose the wall of the vessel and the fluids therein. In contrast to the first cost, this second cost increases with the volume of the network (power associated with metabolism ∼*r*^2^∗*length of the network*). Vessel radius represents a tradeoff between these two costs and determines the total cost to the organism of the vessel network. Minimizing the two power terms leads to a definition of total flux proportional to *r*^3^. This definition can be applied to the special case of a bifurcation where preserving flux and minimizing the amount of power required results in $ r_{0}^{3} = r_{1}^{3} + r_{2}^{3} $. Because we discuss our results in terms of vein diameter rather than radius, we employ an equivalent equation: $ d_{0}^{3} = d_{1}^{3} + d_{2}^{3} $.

Studies of branching architecture have typically found support for Murray’s Law [6]. Murray’s Law was originally developed to describe blood flow in mammals, the system for which the greatest quantity of data is currently available [12, 13]. Murray’s Law also describes fluid flow in various plants [14, 15] despite the vast differences between the circulatory systems of vertebrates and the vascular systems of plants. Vertebrates have a closed circulatory system of fixed volume on short timescales whereas xylem transport is subject to continual volume loss to evaporation, and vertebrate blood contains entire cells whereas plant vasculature transports water and solutes. One of the few commonalities that these transport systems share in plants and vertebrates is unidirectionality within individual conduits. Therefore, circulatory systems with tidal, or oscillatory, flow provide an opportunity to further test the universality of Murray’s Law.

**Fig. 1.**
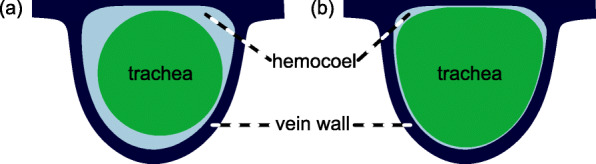
A cross-section of a wing vein of *Attacus atlas*, modified from [18]. In **a**, the trachea does not occupy the entirety of the hemocoel, allowing hemolymph to flow. In **b**, the trachea occupies nearly the entirety of the hemocoel

Although only a few species have been examined to date, the wing veins of obtectomeran Lepidoptera have been found to exhibit tidal flow [16, 17]. Air is displaced with each tracheal contraction, driving flow of both air and hemolymph and allowing more of the cross-sectional area of the vein to be occupied by hemolymph [16] (Fig. 1). This oscillation occurs many times per hour throughout the animal’s adult lifespan [17].

The wing veins of Lepidoptera are far better suited for evaluation of Murray’s Law than the wing veins of other insects for two additional reasons. First, the cross-sectional shapes of wing veins in various insect orders differ markedly from the circular to polygonal shape seen in both plants and vertebrates, which optimizes fluid flow by minimizing cross-sectional area in close proximity to the vein wall [4, 19–22]. The cross-sectional shapes documented in the wing veins of moths are far more circular than those documented in other insect orders [23, 24] and even approach the round shape of vertebrate and some plant veins [16]. This circular cross-sectional shape was recently shown not to be a requirement of Murray’s Law [25] but nevertheless suggests optimization for fluid flow.

Second, the tracheae in insect wings typically occupy less than half of the available area within the veins but nevertheless occupy enough space to disrupt the patterns of hemolymph flow that would occur in their absence [4]. The dimensions of a typical vein cavity, therefore, merely exert an upper limit on the amount of space dedicated to the flow of air and hemolymph—these dimensions constrain but do not determine the transport capacity that Murray’s Law describes. But in Lepidoptera, nearly all space within the vein cavity is dedicated to the flow of air when the tracheae expand, and more space becomes available for the flow of hemolymph when the tracheae contract [16] (Fig. 1).

The dimensions of the vein cavity determine the amount of space dedicated to the flow of both air and hemolymph. The space occupied by a trachea may well violate the assumptions of the Hagen–Poiseuille Law for the hemocoel of a lepidopteran wing vein, thus rendering Murray’s Law inapplicable to the flow of hemolymph. Nonetheless, no such violations of the Hagen–Poiseuille Law are seen within the tracheae themselves. We therefore assume here that the Hagen–Poiseuille Law, and thus Murray’s Law, apply to both the flow of air within the tracheae contained in wing veins and to the flow of hemolymph within wing veins that do not contain tracheae. A study of the atlas moth *Attacus atlas* L. (Saturniidae) found the trachea to occupy nearly the entirety of the vein cavity while expanded [18] such that the interior diameter of the vein determines conformity to Murray’s Law for the flow of air. Critically, none of the mathematical derivations of Murray’s Law [9, 25] assume the flow is from the parent to the child veins; the Law holds equally for reversed flow from the child veins to the parent vein during tidal flow.

**Fig. 2.**
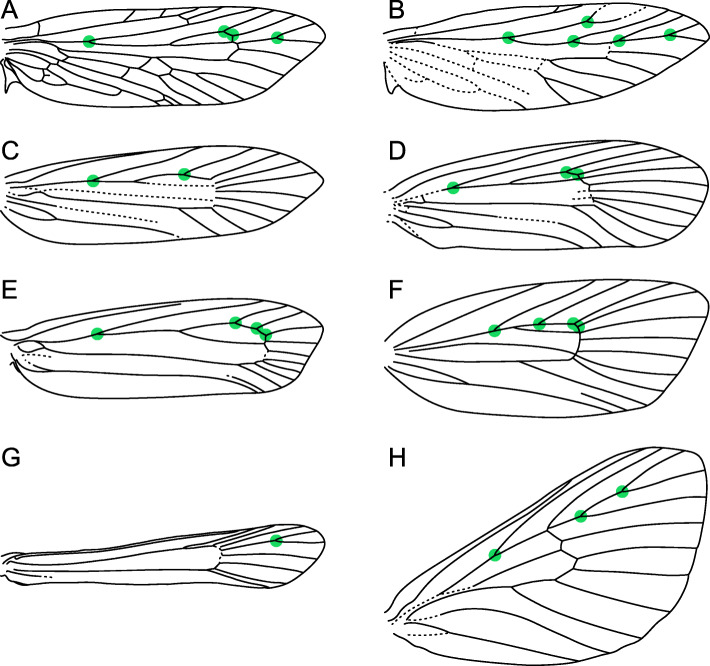
Exemplars of wing venation in lineages within the paraphyletic grade “microlepidoptera.” The green dots represent the bifurcation points that fit the criteria for inclusion in this study; up to three bifurcation points were measured per wing. **A***Agathiphaga vitiensis* Dumbleton (Agathiphagoidea: Agathiphagidae), modified from [26]. **B***Dyseriocrania* Spuler (Eriocranioidea: Eriocraniidae), modified from [27]. **C***Incurvaria masculella* Haworth (Adeloidea: Incurvariidae), modified from [28]. **D***Scardia anatomella* Treitschke (Tineoidea: Tineidae), modified from [29]. **E***Yponomeuta* Latreille (Yponomeutoidea: Yponomeutidae), modified from [30, 31]. **F***Argyroploce* Hübner (Tortricoidea: Tortricidae), modified from [32]. **G***Pennisetia marginata* Dehne (Sesioidea: Sesiidae), modified from [33]. **H***Pseudanapaea trigona* Hering (Zygaenoidea: Limacodidae), modified from [34]

**Fig. 3.**
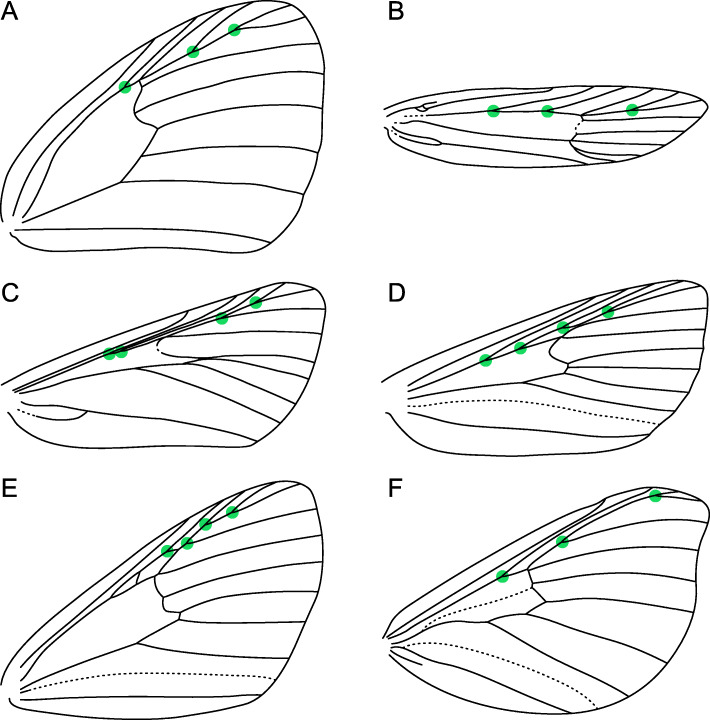
Exemplars of wing venation in younger superfamilies. The green dots represent the bifurcation points that fit the criteria for inclusion in this study; up to three bifurcation points were measured per wing. **A***Physcaeneura panda* Wallengren (Papilionoidea: Nymphalidae), modified from [35]. **B***Dichomeris marginella* Hübner (Gelechioidea: Gelechiidae), modified from [36]. **C***Cacozelia basiochrealis* Grote (Pyraloidea: Pyralidae), modified from [37]. **D***Acronicta lobeliae* Guenée (Noctuoidea: Noctuidae), modified from [38]. **E***Hydriomena costipunctata* Barnes and McDunnough (Geometroidea: Geometridae), modified from [39]. **F***Neocercophana philippii* Izquierdo (Bombycoidea: Saturniidae), modified from [40]

Here we present an evaluation of Murray’s Law for the wings of hundreds of species spanning the entire lepidopteran phylogeny. These wings vary in size, shape, and in the branching architecture of the venation (Figs. 2 and 3), permitting a nuanced perspective on the selective pressures that may underlie vein optimization for fluid transport. This study is both confirmatory and exploratory. It is confirmatory in testing the hypothesis that Murray’s Law describes the geometry of vein branching patterns in Lepidoptera. It is also exploratory in assessing the extent to which conformity to Murray’s Law varies as a function of taxonomic affiliation and parent vein diameter—relationships for which there is no pre-existing hypothesis to test.

## Results

We found that Murray’s Law consistently predicts branching architecture for bifurcations in which the parent vein has a diameter above 45–58 microns. For smaller veins, Murray’s Law has less and less predictive power as diameter decreases. Bifurcation angle, unlike vein diameter, does not predict conformity to Murray’s Law. A sensitivity analysis found that the images used are of sufficient pixel density for precise measurements of the diameters of vein cavities (Additional file 1: Figures S1, S2).

Murray’s Law was evaluated here by solving for *k* in the equation outlined above. Because the veins of moth wings bifurcate into two child veins, the appropriate representation of the equation is: $ d_{0}^{k} = d_{1}^{k} + d_{2}^{k} $. The bifurcations that most closely conform to Murray’s Law are those for which *k* is closest to 3.

**Fig. 4.**
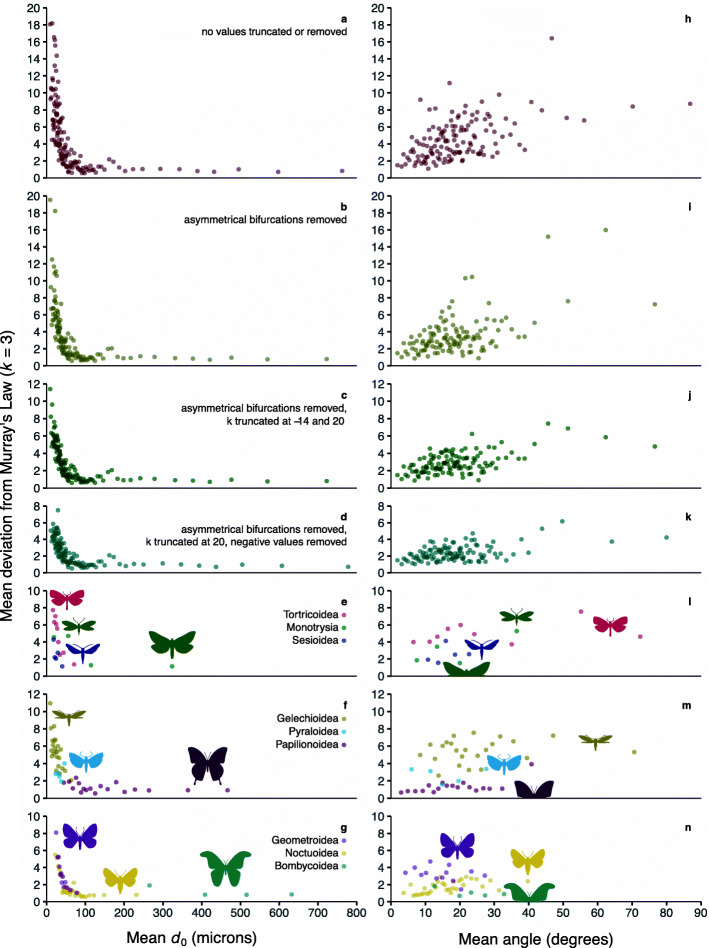
The relationships between *d*_0_ and bifurcation angle with *k* illustrating that *k* is highly variable at small *d*_0_ and converges on values near 3 (Murray’s Law) at larger *d*_0_ and that *k* progressively diverges from 3 at larger bifurcation angles. For ease of comprehension, each point in the scatterplots represents the average value for twenty observations. Bifurcations were sorted by *d*_0_ (**a**–**g**) and angle (**h**–**n**) before being binned into groups of twenty. The raw data are presented in the supplemental figures. Note that because panels a–g illustrate the absolute deviation from *k* = 3, the mean deviation will not reach zero simply due to measurement error or any small deviation

The values of *k* observed in Lepidoptera vary tremendously (Fig. 4; Additional file 2: Figure S3). Of the 2696 bifurcations examined, 127 have child veins that are both wider than the parent vein, yielding a negative value of *k*. Another 338 bifurcations have one child vein that is wider than the parent vein and one that is narrower than the parent vein. Some bifurcations with this type of asymmetry yield values of *k* consistent with the various biophysical predictions outlined above, while others yield values of *k* below − 100 or above 70. Both child veins are narrower than the parent vein, as predicted by Murray’s Law and other theoretical optima, in 83% of the bifurcations examined.

**Fig. 5.**
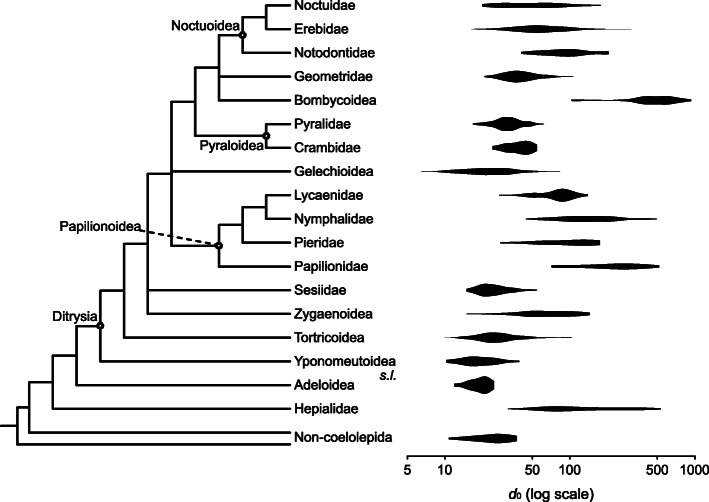
The relationships among the superfamilies examined here, and the ranges of *d*_0_ (measured in microns) in each. The superfamilies that have been divided into families are those that contain at least two families with at least 20 measurements of *d*_0_ in our dataset and whose monophyly is well-established. Note that the ranges of *d*_0_ observed in Noctuidae, Erebidae, Geometridae, and Zygaenoidea overlap with the ranges of *d*_0_ observed in all other families and superfamilies in this figure

But despite the sensitivity of *k* and the variability in the dataset, and regardless of how the dataset is processed—whether or not negative values of *k* and asymmetrical bifurcations are included and whether or not *k* is truncated—a clear pattern emerges (Fig. 4a–d). The average deviation from Murray’s Law (*k* = 3), defined simply as |*k*−3|, decreases as the cross-sectional diameter of the parent vein (*d*_0_) increases—until *d*_0_ falls within the range of 45–58 microns (the 95% confidence interval spans diameters as low as 39 microns and as high as 65 microns). Beyond this point, deviation from Murray’s Law remains low as *d*_0_ continues to increase with the slope of this segment nearly equal to 0—ranging from − 0.0018 to − 0.0007—and the 95% confidence interval for the slope always includes 0. Visualization of our results in phylogenetic context demonstrates that our findings are not merely an artifact of phylogenetic autocorrelation (Fig. 5; Additional file 3: supplement 2).

### Vein angle

In contrast to *d*_0_, the relationship between vein angle and *k* is not clear and may be an artifact of the phylogenetic relationships among the lineages sampled (Fig. 4h–n; Additional file 4: Figure S4). In the four datasets that contain all species, *k* conforms closely to Murray’s Law in the bifurcations for where the two child veins connect at a very narrow angle, below approximately five degrees (Fig. 4h–k). *k* is more variable in the bifurcations with wider angles. The few bifurcations with angles greater than 40^∘^ tend to yield values of *k* that deviate strongly from Murray’s Law. However, unlike *d*_0_, vein angle appears to have a relationship with *k* that is largely an artifact of the phylogenetic relationships among the taxa sampled, as shown by the stratification of superfamilies within higher grades and clades (Fig. 4l–n). Vein angle varies widely in smaller moths but does not exceed 30^∘^ in larger moths, further supporting the notion that selective pressures increase with vein diameter.

**Fig. 6.**
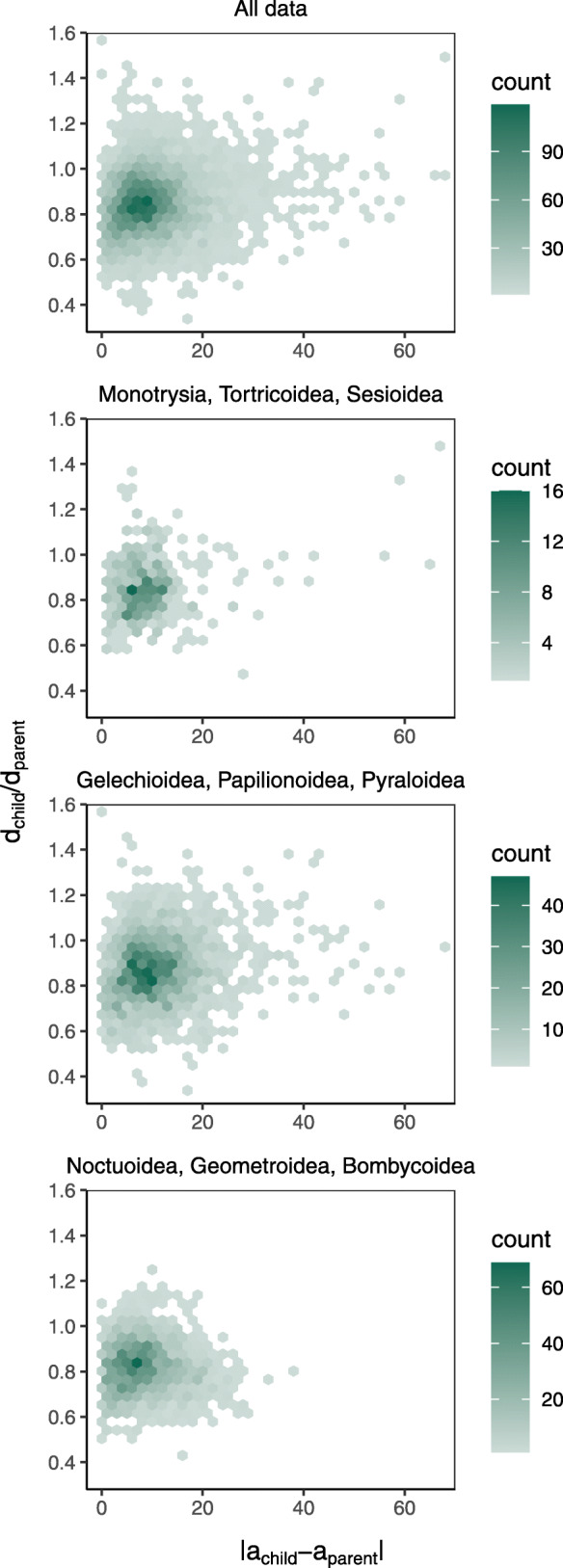
Heatmaps showing the differences in angle (mean value: 10.1076^∘^, standard deviation: 7.3398^∘^) and diameter (mean value: 0.8496 microns, standard deviation: 0.1289 microns) between all pairs of parent and child veins measured here. The value along the *x*-axis is the difference in angle between each parent and child vein. For example, if a wing is rotated so that the parent vein is at an angle of 0^∘^ and one of its child veins is at an angle of 30^∘^, the value for this pair of parent and child veins along the *x*-axis will be 30

The relationship between vein angle and fluid transport can also be evaluated by comparing the angle of each individual child vein to the angle of its parent vein, which can vary noticeably even when the angle between the child veins is small. Murray himself predicted that, when two child branches are of unequal diameter, the wider child branch will occur at a lower angle to the parent branch [9, 41]. However, in Lepidoptera, there is no trend in the relationship between the difference in angle between the parent and child vein and the difference in diameter (Fig. 6).

### Trichoptera: the sister-group to lepidoptera

**Fig. 7.**
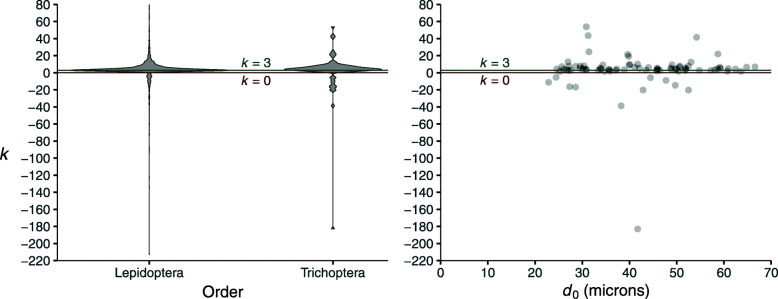
Left: Violin plots comparing the values of *k* in Lepidoptera and Trichoptera. Right: the relationship between *k* and *d*_0_ in Trichoptera

The distribution of *k* among 82 bifurcations in the R vein on the wings of Trichoptera is very similar to the distribution seen in Lepidoptera (Fig. 7). However, the relationship between *k* and *d*_0_ in Trichoptera is unclear (Fig. 7).

This relationship does not show the same size-dependence in Trichoptera as in Lepidoptera. When the same change-point analysis discussed above was implemented for Trichoptera, the 95% confidence interval recovered for the value of *d*_0_ at the change point (23–64 microns) was nearly identical to the entire range of *d*_0_ in the dataset (23–67 microns). Furthermore, the 95% confidence intervals for the slope both before and after the change point include 0. It is unclear whether additional data, perhaps spanning a wider range of *d*_0_, would show a discernible pattern or a biological reason for this deviation.

## Discussion

Murray’s Law predicts branching architecture reasonably well in the wings of Lepidoptera. This finding of a shared biophysical optimum among the circulatory and vascular systems of Lepidoptera, vertebrates, and various plants suggests that Murray’s Law is one of the few theoretical predictions that holds throughout the multicellular tree of life—at least at the diameters examined thus far.

The primary caveat to the applicability of Murray’s Law in Lepidoptera is vein diameter: smaller moths, whose parent veins have diameters below 45–58 microns, are the least likely to conform to Murray’s Law. The circulatory systems in the wings of these moths do not conform to a different biophysical optimum, and instead evince a far wider range of vales of *k* than is seen in larger moths. This suggests that circulatory systems in the wings of smaller moths are not under distinct selective pressures to those that determine vein morphology in larger moths, but instead are under weaker selective pressures.

The importance of hemolymph transport in the wings of Lepidoptera, particularly of small moths, is not entirely understood. In this order, hemocytes appear to occur at far greater abundances in larvae than in adults; although many hemocytes can be seen in the hemolymph circulating throughout the wing veins of the butterfly *Vanessa cardui* L. [17], data on hemocyte quantity in the wings of microlepidoptera are lacking, and the wing veins of many microlepidoptera may be too narrow to transport hemocytes. However, studies of Diptera show that hemolymph can continue to flow through wing veins even when the diameter of the vein cavity is too small to accommodate hemocytes [42, 43]. The importance of hemolymph transport in microlepidoptera, regardless of hemocyte content, is underscored by the widespread presence in Monotrysia of accessory pulsatile organs known as “wing hearts” [44]. Because these organs pump hemolymph into wing veins [45], their presence in small-bodied, early-diverging families such as Eriocraniidae and Lophocoronidae indicates that the hemolymph flow in these small moths is of sufficient physiological importance to require dedicated anatomical structures—although we do not have sufficient data from Lepidoptera to determine whether the cells that line vein walls [4, 46] conform to the assumptions about the cost function of Murray’s Law.

The relaxation of selective pressures for animals with smaller body sizes has been noted in other taxa. When digits first originated in tetrapods their numbers varied tremendously but then stabilized very quickly [47]. Digit reduction and loss among extant taxa occurs frequently but predictably, in terms of both life history and the digits affected [48, 49]. This consistency often disappears, however, among the smallest-bodied salamander and frog genera, in which digits can become reduced and lost in a highly variable and seemingly random manner [50–52].

Lepidoptera are hardly the only clade that possesses a size threshold that separates strong and weak selective pressures. Weaker selective pressures at small body sizes are particularly common for gas exchange because diffusion becomes an adequate method of gas exchange upon sufficient reduction of body size [53].

**Fig. 8.**
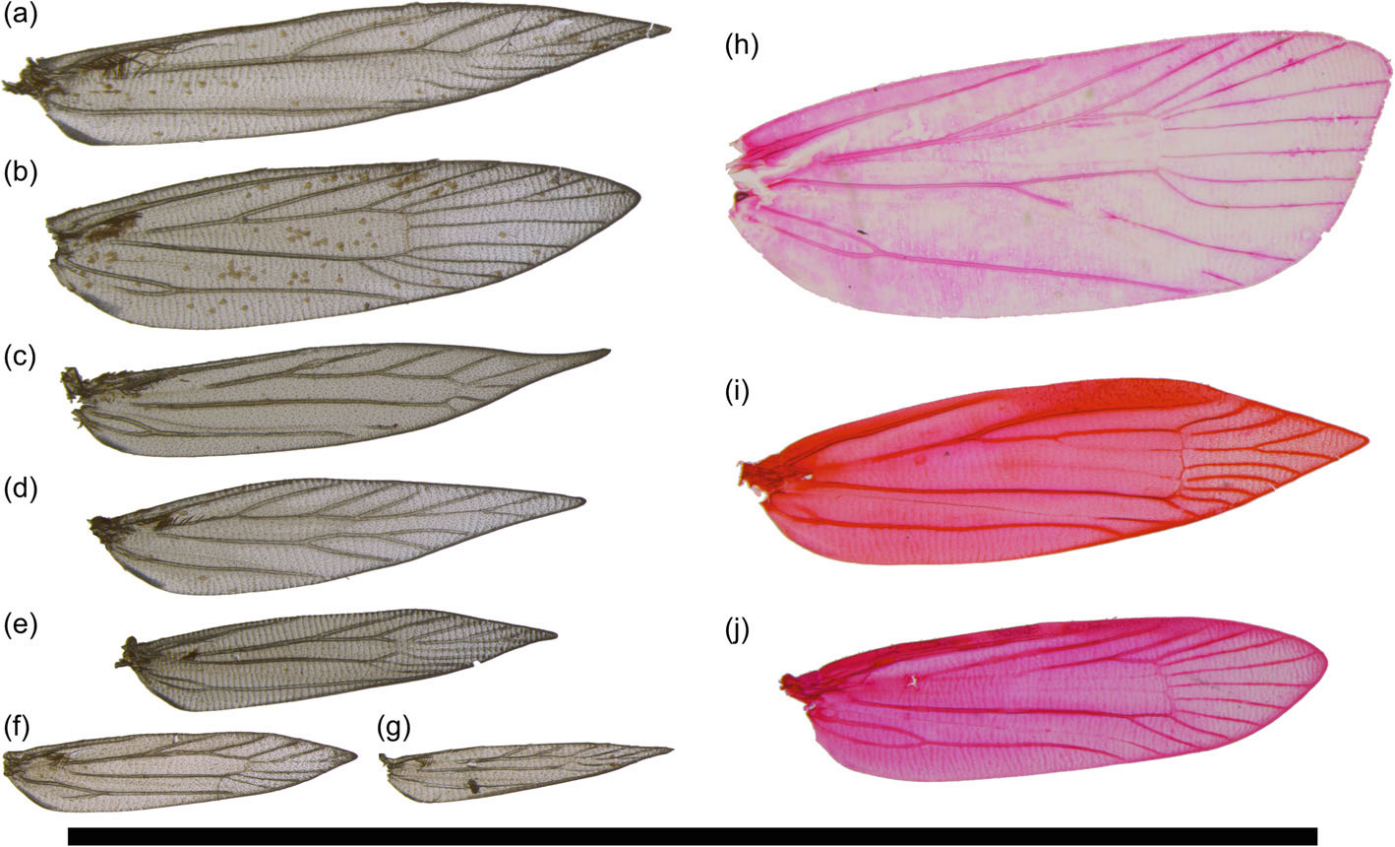
Wing slides showing venation in various microlepidoptera. **a***Aetia bipunctella* Chambers (Gelechioidea: Elachistidae; USNM 86446). **b***Triclonella pergandeella* Busck (Gelechioidea: Momphidae; USNM 86421). **c***Coleophora* Hübner sp. (Gelechioidea: Coleophoridae; USNM 86413). **d***Theisoa multifasciella* Chambers (Gelechioidea: Gelechiidae; USNM 86510). **e***Ithome concolorella* (Chambers) (Gelechioidea: Cosmopterigidae; USNM 86431). **f***Ymeldia janae* Hodges (Gelechioidea: Oecophoridae; USNM 86514). **g***Melanocinclis lineigera* Hodges (Gelechioidea: Cosmopterigidae; USNM 86416). **h***Larisa subsolana* Miller (Tortricoidea: Tortricidae; USNM 71795). **i***Argyresthia alternatella* Kearfott (Yponomeutoidea: Argyresthiidae; MEM 2985B). **j***Tinea apicimaculella* Chambers (Tineoidea: Tineidae; MEM 3025B). Scale bar: 1 cm

**Fig. 9.**
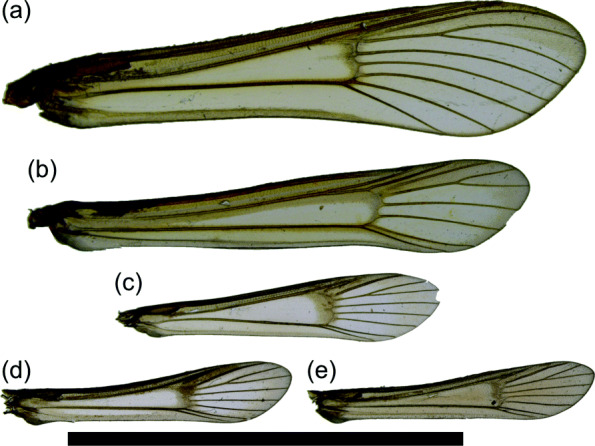
Wing slides showing venation in Sesiidae. **a***Sesia apiformis* Clerck (USNM 75815). **b***Pennisetia hylaeiformis* Laspeyres (USNM 75829). **c***Carmenta corni* Edwards (USNM 75678). **d***Carmenta albociliata* Engelhardt (USNM 75675). **e***Carmenta verecunda* Edwards (USNM 75680). Scale bar: 1 cm

In small moths, the decreased importance of the circulatory function of wing veins is readily apparent: veins regularly atrophy, both at the base and distally to the discal cell (Fig. 8). The wing veins of Lepidoptera are said to “atrophy” when they seemingly disappear from the wing [54], becoming visible only as “faint traces” if at all [44]. “Atrophied” veins were present in the developing wings of the pupa but are not intact in the adult wings. Although it cannot be stated with complete certainty that atrophied veins do not transport any fluid in the adult wing, their morphology is not optimized for fluid transport. The role of wing veins in circulation is limited even in small moths whose veins do not atrophy because much of the wing surface area used for flight derives from the wing fringe. The fringe consists only of scales, contains no living cells, occurs beyond the wing membrane, and therefore does not contain any veins. Furthermore, when the vein of a small moth bifurcates, the trachea within it often does not, and may not even extend far enough from the wing base to reach the point where the vein bifurcates [55, 56]. Furthermore, we note that many of the microlepidoptera examined here whose values of *k* deviate strongly from Murray’s Law have atrophied veins and veins that only appear at the wing margin (Figs. 8 and 9).

Thus, conformity to Murray’s Law in many lepidopteran taxa cannot be attributed solely to optimization for fluid transport in the adult wing. However, in addition to minimizing the pump work required to transport fluid, a network analysis by [57] has shown that vein networks adhering to Murray’s Law have minimal total mass for a given flow transport rate. And as detailed in the supplemental material (Additional file 5), Murray’s Law also describes a structural optimum for Lepidoptera whose wings are not corrugated. This provides a potential explanation for conformity to Murray’s Law, and its size-dependence, in taxa such as Sesiidae whose veins atrophy before bifurcating. Alternatively, conformity to Murray’s Law in moths with atrophied veins on the adult wing may be an artifact of optimization for fluid transport during the pupal stage, before veins reach the atrophied state seen in the adult wing. Heightened conformity to Murray’s Law during earlier stages of development has been observed in humans [58] and could be evaluated for Lepidoptera in future studies that examine pupal wings.

In the human circulatory system [11, 59–61], bifurcations follow da Vinci’s Law more closely than Murray’s Law at larger diameters. For all 455 bifurcations measured here in which *d*_0_ is at least 100 microns—a threshold at which conformity to Murray’s Law has definitely stabilized—the mean value of *k* is 2.91 (95% confidence interval: 2.75–3.09). Although *k* does not exactly equal 3, this value very nearly falls within the range observed in pigs [59] that provides strong empirical support for Murray’s Law. However, for the 100 bifurcations with the largest values of *d*_0_, ranging from 360 to 932 microns, the mean value of *k* is 2.51 (95% confidence interval: 2.35–2.69). Future studies that examine venation in larger individuals belonging to the species *Attacus atlas* and in other large-bodied species such as *Thysania agrippina* (Cramer) (Erebidae) could provide greater insight into whether the wings of Lepidoptera conform more closely to da Vinci’s law at larger vein diameters.

## Conclusions

Lepidoptera are the most diverse taxon for which the applicability of Murray’s Law has been examined. Our findings demonstrate the size-dependent relevance of Murray’s Law to Lepidoptera, a conclusion that is robust to the evolutionary relatedness of the taxa sampled. Some of our findings, such as the potential relevance of da Vinci’s Law to the largest vessels, are consistent with previous studies of both mammals and plants. However, our dataset also includes novel features such as the sharp decrease in conformity to Murray’s Law in vessels below 50 microns and the relationship between Murray’s Law and vessel size even for bifurcations that occur distally to atrophied veins. Our findings highlight the universality of Murray’s Law and raise questions about the selective pressures that may underlie its applicability.

## Methods

The cross-sectional widths of the internal cavities of lepidopteran forewing veins were measured from microscope slides of “cleared” wings, from which the scales have been bleached and removed to facilitate visualization of veins. Measurements were taken from the radius vein (*R*), which in moths bifurcates into branches known as *R*_1_,*R*_2_,*Rs*_1_,*Rs*_2_,*Rs*_3_, and *Rs*_4_ [24]. This vein was chosen because it typically bifurcates at least three times before connecting with branches of the medial vein via cross-veins. Forks in *R* were only measured if both of the resulting branches reach the margin of the wing, such that forks whose posterior branch forms a closed cell were not measured. Forks could not be measured when the branching point occurred immediately distal to the confluence of two veins. The points that were measured are illustrated on the examples provided in Figs. 2 and 3. Wings whose *R*vein bifurcates only twice were not included in this study unless those wings are less than six millimeters in length. All wing slides in the dataset were photographed in the collections where they are housed or were taken on loan and photographed at Stanford University with the exception of the ctenuchine and euchromiine wing slides, which were not available for loan and could only be measured from photographs.

Of the primary veins on the forewings of Lepidoptera, the subcosta branches in some homoneura [27, 62] and in Arrhenophanidae [63] but is unbranched in the vast majority of species, the cubitus posterior vein branches in exceedingly rare cases [26], and the anal veins are variable in microlepidoptera [27, 28, 62] and often lack any confluence or bifurcations in butterflies [35] and Macroheterocera [40, 64, 65]. This leaves the radius, medial, and cubitus anterior as the only veins with which Murray’s Law can be evaluated across Lepidoptera. The radial sector typically branches into *Rs*_1_,*Rs*_2_,*Rs*_3_, and *Rs*_4_, permitting multiple measurements from the same vein. The branching patterns of the medial and cubitus anterior veins are far less consistent: the medial vein contains up to five branches [26], with three being the typical number outside *Agathiphaga*—with the crucial exception of Noctuoidea, the lepidopteran superfamily with the greatest described species diversity, in which countless “quadrifid” species have four branches of the medial vein [64]. Complicating matters further, free *M* and *CuA* veins both reach the base of the wing in various microlepidoptera but not in Obtectomera (Figs. 2 and 3). As noted by [66], this variation causes meaningful differences in hemolymph circulation.

### Measurements

In each forewing, the *R* vein bifurcates at least once. Data were collected here for up to three points of bifurcation. At each point of bifurcation, the vein diameter was measured for all three components, the parent and two child branches. Along with a measurement of overall wing length, this resulted in four, seven, or ten measurements per wing, depending on whether one, two, or three points of bifurcation were measured. The branching pattern of the *R* vein varies tremendously among moths (Figs. 2 and 3), often but not always as a result of different numbers of total branches of this vein. Four measurements were taken for Sesiidae: *R* atrophies (seems to disappear because the vein walls disintegrated during the pupal stage) in sesiids at the most proximal points where *R* typically bifurcates, with the exceptions of two genera examined here, *Bembecia* Hübner and *Melittia* Hübner. In these genera, the cross-sectional diameter of *R* increases consistently until it first bifurcates, such that there is no optimal location for measuring this diameter. This same set of four measurements was also taken for Ctenuchina + Euchromiina because the first point of bifurcation in *R* was the only one that could be consistently measured from the photographs provided.

**Fig. 10.**
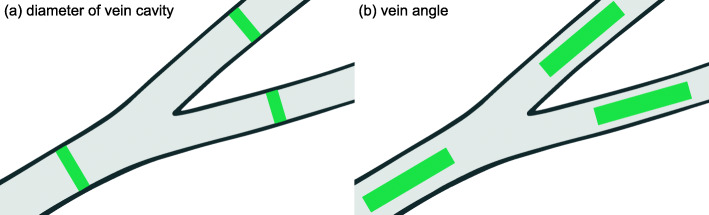
A schematic of the second bifurcation on the wing of *Diloba caeruleocephala* L. showing how the diameter of the vein cavity was measured (**a**) and how the vein angles were measured (**b**)

Hemolymph is known to leak out of the veins into the living tissue of the surrounding wing lamina [67], and the veins taper along the proximo-distal axis of the wing. To minimize the impact of vein tapering and leakage on the measurements, veins were measured as close as possible to the points where they bifurcate, as [68] have done. Here, all branches corresponding to the radius and radial sector (*Rs*) veins in Wootton’s terminology [69] are treated as branches of *R* because all originate from the main *R* vein. A schematic of how we made our diameter and angle measurements is presented in Fig. 10.

All available wing slides from the Mississippi Entomological Museum and the Finnish Museum of Natural History were examined. The Lepidoptera slide drawers at the Smithsonian Institution that have been assigned United States National Museum numbers, which have the capacity to hold 155,548 slides, were searched for wing slides. All slides made with non-type material—i.e., slides made from specimens that have not been designated as name-bearers for their species—from the USA were taken on loan. This sampling method is unbiased in that all available material was examined, but the resulting dataset is taxonomically biased toward the taxa for which wing slides are typically made. Wing slides of zygaenoid moths housed in the California Department of Food and Agriculture were examined. Photographs of three wing slides of *Agathiphaga vitiensis* Dumbleton, taken for a recent study [26], were included. These photographs are of sufficient pixel density to measure the widths of veins and were included because of the phylogenetic position of this taxon. Photographs of wing slides of Ctenuchina + Euchromiina were included because this taxon is not otherwise represented in the dataset. All of these wing slides were created for previous taxonomic and morphological studies [26, 70–82].

When these wing slides had been examined, the superfamilies Hepialoidea, Papilionoidea, and Bombycoidea were noticeably underrepresented in the dataset, with zero, one, and three wing slides, respectively. New wing slides for various species of Hepialidae were made from specimens at the EMEC (Essig Museum Entomological Collection, University of California, Berkeley) and the CASC (California Academy of Sciences). New wing slides for the hepialid species *Gazoryctra mcglashani* (Edwards) were made from two specimens provided by Laurence L. Crabtree, one of the two lepidopterists who recently documented re-encountering this species [83]. New wing slides for Papilionoidea and Bombycoidea were made from museum specimens from the EMEC and CASC and with specimens from the rainforest exhibit at the CAS that were collected after they perished. New wing slides for the atlas moth were made with specimens collected by Joshua Cluck at the Butterfly Habitat in Vallejo, California, after they perished.

These same measurements were also made from the wings of caddisflies (order Trichoptera), the sister group to Lepidoptera. The species examined were assigned to the subordinal clades identified by [84]. Rhyacophilidae, a family of controversial affinities, is the only representative in the dataset of the group traditionally referred to as Spicipalpia and now known as Integripalpia *sensu* [84]. In order to avoid confusion in the wake of recent changes to caddisfly taxonomy, this family is not discussed here as a representative of any higher clade. Only two measurements were made from each wing because the first point at which *R* bifurcates typically occurs very close to the base of the wing, where the edges of the veins are difficult to discern. Spread caddisflies from the EMEC and CASC were photographed for measurement at the EMEC.

Some caddisfly species, particularly belonging to the brevitentorian superfamily Leptoceroidea [84], have forewings with such a dense covering of hair that the veins can only be seen if the wings are removed and the hairs are brushed off—the same process used to create wing slides for Lepidoptera. One forewing was removed from three specimens each of *Phylloicus aeneus* (Hagen) and *Heteroplectron californicum* McLachlan (Calamoceratidae), and *Namamyia plutonis* Banks (Odontoceridae), at the EMEC. The wings broke apart when immersed in 70% ethanol, making it very difficult to remove the hairs before the wings became fragmented. Therefore, measurements of wing vein diameter could only be taken from two of the nine wings prepared.

We measured the diameters of vein cavities with photomicrographs that have a density of 146–4,660 pixels per millimeter depending on the size of the wing (mean value: 2,022 pixels/mm, 4,088,484 pixels/mm^2^). These photomicrographs were taken at Stanford University with a Leica M165 C microscope and a Leica DFC450 camera, at the Finnish Museum of Natural History (Luomus) with a Leica DM1000 LED and Leica S9D camera, and at the EMEC with a Leica S9i microscope and built-in camera. The slides were backlit with a stage light. With this optical setup, the boundary between the vein cavity and the vein wall is readily discernible. The widths were measured using the Pen Tool in Affinity Designer version 1.8.3, with the default Round Cap changed to the Butt Cap. The dimensions of each line were extracted from the Transform Panel in Affinity Designer and pasted into a Microsoft Excel spreadsheet.

### Systematics

**Fig. 11.**
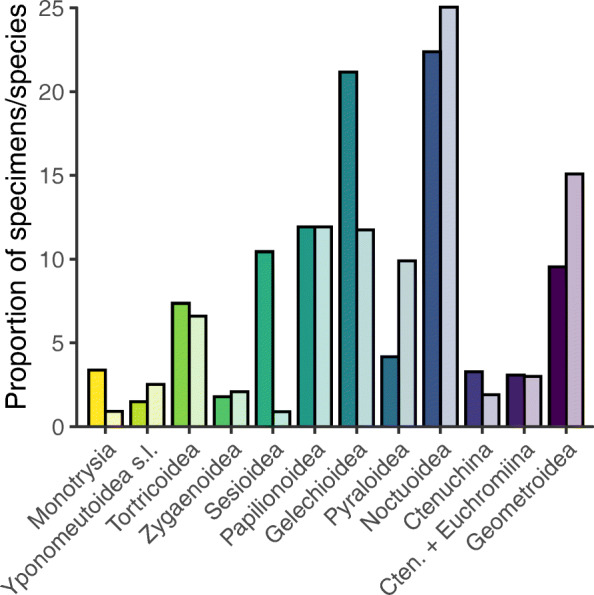
Sampling by higher taxon. Relative proportions of the wings sampled for this study, on the left in dark colors, compared to their relative proportions of described species, on the right in light colors [85]. The only superfamilies included in this graph and in the underlying calculations are those represented by three or more specimens in this study. All Monotrysia were lumped into a single category. “Cten.” is an abbreviation for Ctenuchina

The representation of moth superfamilies among the specimens examined here roughly parallels the described species-level diversity of those superfamilies (Fig. 11). Gelechioidea, the most speciose superfamily of microlepidoptera [85], is noticeably overrepresented in the dataset. This superfamily is noted for its high proportion of undescribed species and may contain more true species-level diversity than any other superfamily in the order [86].

For the expository figure showing sampling effort, for the figures plotting various metrics against wing length, and for the linear regressions comparing these metrics to wing length, the paraphyletic grade Monotrysia, which contains the most early diverging superfamilies of extant Lepidoptera, was treated as a single superfamily. Although hepialid anatomy is exoporian rather than monotrysian [27], Hepialidae were included in the Monotrysia because they diverged within this grade, i.e., they diverged before the Ditrysia [85]. Monotrysia were lumped together because many of the monotrysian superfamilies contain a single family [85]. Because the superfamily Gracillarioidea is represented by fewer than 10 specimens in the dataset, and because this superfamily is suspected to be paraphyletic with respect to Yponomeutoidea [87], Gracillarioidea, and Yponomeutoidea were combined into a “Yponomeutoidea s.l.” taxon. Because no explicitly phylogenetic analyses were conducted for this study, and because all analyses of individual superfamilies were conducted separately, these decisions to lump superfamilies did not impact any of the results.

The subtribes Ctenuchina and Euchromiina belong to the superfamily Noctuoidea [88]. However, because Ctenuchina and Euchromiina are unique among Noctuoidea in that they often have clear wings, and because they are sister taxa that form a monophyletic clade [89], we treat them as a separate group here. “Noctuoidea” is used here to describe all noctuoid moths except for Ctenuchina + Euchromiina.

### Figures and analyses

For the main scatterplot illustrating the values of *k* found in this study, each point represents the mean value for 20 bifurcations (before the data were binned into groups of 20 bifurcations, they were arranged by the variable of interest, either *d*_0_ or angle). This decision was made because plots of the raw data (i.e., values of *d*_0_, bifurcation angle, and *k* for individual bifurcations) are difficult to comprehend—not only because of the size of the dataset, but because the wide range of values of *k* precludes differentiation between *k* = 2, *k* = 3, etc. (Additional file 2: Figure S3). The decision to plot mean values for groups of 20 bifurcations vastly reduced the range of the data, permitting inferences at a much finer scale. Of the 338 bifurcations in which one child vein is wider than the parent vein and one is narrower than the parent vein, three have values of *k* that are so negative that they cannot be calculated with the software used (R version 4.0.2), and thus were removed from the dataset.

Four datasets are presented in the main scatterplot, with different kinds of processing. The first dataset is unprocessed, with nothing truncated or removed. In the second dataset, asymmetrical bifurcations (in which one child branch is wider than the parent and the other is narrower) were removed. In the third dataset, asymmetrical bifurcations were removed and values of *k* were truncated at − 14 and 20. In the fourth dataset, asymmetrical bifurcations were removed, bifurcations in which both child branches are wider than the parent were removed, and *k* was truncated at 20.

Analyses were performed on the raw data rather than the truncated or binned data. To evaluate the *d*_0_ threshold at which conformity to Murray’s Law changes, change point analysis was performed with the mcp package version 0.3.0 [90] in R version 4.0.2 [91] with the default settings (Gaussian family, 9000 iterations from 3 chains). A generalized linear model for the effects of vein diameter and microscope magnification was performed on the data collected at the Finnish Museum of Natural History (Luomus), with magnification coded as a quantitative variable represented by the number of pixels corresponding to one millimeter.

## Supplementary Information

**Additional file 1** Supplement 1. Supplementary methods, results, and discussion of the potential impact of image quality. **Figure S1.** Values of *k* for bifurcations measured at the Finnish Museum of Natural History (Luomus), categorized by the magnification of the microscope when the wing slides were photographed. **Figure S2.** Measurement error, in microns, of the diameters of 100 wing veins that were measured twice at with the same microscope at the same magnification.

**Additional file 2Figure S3.** The raw relationship among *d*_0_ and *k*.

**Additional file 3** Supplement 2. Supplementary discussion of phylogenetic context.

**Additional file 4Figure S4.** The raw relationship among bifurcation angle and *k*.

**Additional file 5** Supplement 3. Supplementary discussion of generalization of Murray’s Law for insect wings under flow transport, structural function, and total vein network mass constraints.

## Data Availability

All data and code are available at https://purl.stanford.edu/wq177gw5815.
