# Supplementary material for: Lepidoptera demonstrate the relevance of Murray’s Law to circulatory systems with tidal flow
Source: BMC Biol. 2021 Sep 15;19:204. doi: 10.1186/s12915-021-01130-0 (PMC8444497; doi:10.1186/s12915-021-01130-0)
Supplement: Supplementary file 1 — Additional file 1 Supplement 1. Supplementary methods, results, and discussion of the potential impact of image quality. Figure S1. Values of k for bifurcations measured at the Finnish Museum of Natural History (Luomus), categorized by the magnification of the microscope when the wing slides were photographed. Figure S2. Measurement error, in microns, of the diameters of 100 wing veins that were measured twice at with the same microscope at the same magnification. [file 12915_2021_1130_MOESM1_ESM.pdf]

# Additional file 1 for: Schachat et al. Lepidoptera demonstrate the relevance of Murray's Law to circulatory systems with tidal flow in *BMC Biology*

# The potential impact of image quality

If all bifurcations were photographed at the same magnification, the variability of $k$ at smaller vein diameters described here might be an artifact of the pixel density of each vein in the photographs from which diameters were measured. However, the magnification of the microscope was adjusted for each slide while the pixel density of the resulting image remained constant. To confirm that the values of $k$ reported here are not artifacts of microscope magnification, we examined the relationship between $k$ and microscope magnification for all measurements taken at the Finnish Museum of Natural History (Luomus). The bifurcations measured at Luomus were chosen for this exercise because they were photographed with a slide microscope at one of three magnifications: 10x, 20x, or 40x.

A scatterplot of $k$ color-coded by magnification does not suggest that bifurcations photographed at lower magnification yield values of $k$ that deviate more strongly from Murray's Law (Figure S3a). This observation was verified with a Kruskal–Wallis rank sum test of values of $k$ from bifurcations in which $d_0$ is between 25 and 35 microns, a size range at which 22–61 bifurcations were photographed at each magnification. This test returned a marginally insignificant result ($p = 0.089$). The potential impact of magnification can be further interrogated by examining a violin plot and box plot of $k$ at this size range (Figure S3b). This plot shows that bifurcations photographed at low magnification deviate from Murray's Law less than bifurcations photographed at high magnification, contradicting the expected result under a scenario in which deviations from Murray's Law at lower vein diameters are an artifact of image quality.

This finding can be explained by the fact that all bifurcations on a wing were photographed at the same magnification, which was constrained by the diameter of the widest $d_0$ on the wing. Therefore, the bifurcations for which $25 < d_0 < 35$ that were photographed at low magnification are most likely to be the first, or most proximal, bifurcation on the wing, and bifurcations for which $25 < d_0 < 35$ that were photographed at medium or high magnification are more likely to occur more distally. And because proximal bifurcations tend to have a wider $d_0$, they are less likely to deviate from Murray's Law.

Because magnifications were not applied randomly with respect to $d_0$, these data were analyzed with a generalized linear model which evaluates the mean deviance from Murray's Law as a simultaneous function of $d_0$ and magnification. The model confirms that the effect of magnification after accounting for $d_0$ is not significant ($p = 0.45$) but the effect of $d_0$ after accounting for magnification is ($p = 0.0002$). Therefore, the values of $k$ presented here are not biased by the magnification at which photos were taken.

To estimate the amount of error in the raw measurements, we conducted repeated measurements of the diameter of the main R vein on 100 specimens. We photographed this vein on each specimen, then re-set the microscope by adjusting and readjusting the lighting and focus, and then photographed the vein again. We measured the diameter of the vein from each photograph and then calculated the difference, in microns, between the two measurements for each specimen. Our results show that measurement error is minimal, ranging from 0.00 to 1.79 microns (Figure S4). The mean error is 0.45 microns and the bootstrapped 95 % confidence interval (calculated with 10,000 iterations) ranges from 0.38 to 0.52.

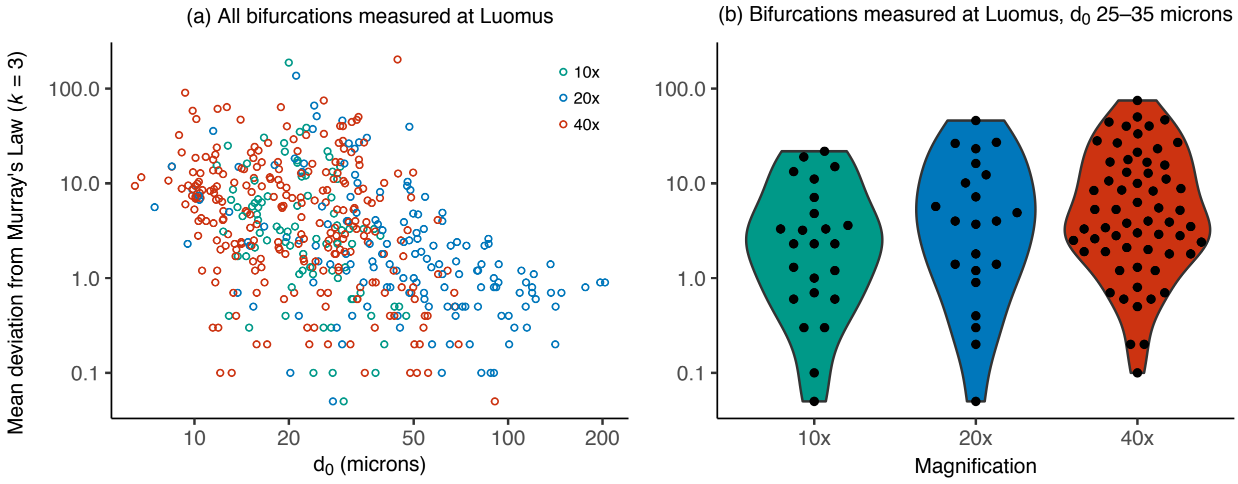


Figure S3: Values of $k$ for bifurcations measured at the Finnish Museum of Natural History (Luomus), categorized by the magnification of the microscope when the wing slides were photographed.

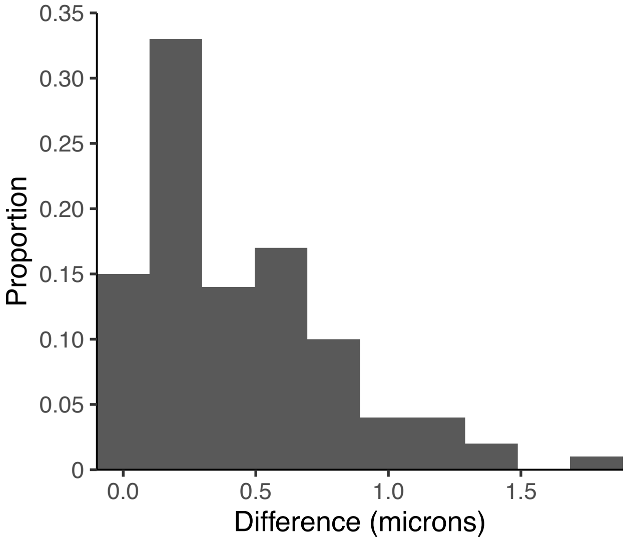


Figure S4: Measurement error, in microns, of the diameters of 100 wing veins that were measured twice at with the same microscope at the same magnification.
