# Supplementary figures and images for: Lepidoptera demonstrate the relevance of Murray’s Law to circulatory systems with tidal flow
Source: BMC Biol. 2021 Sep 15;19:204. doi: 10.1186/s12915-021-01130-0 (PMC8444497; doi:10.1186/s12915-021-01130-0)

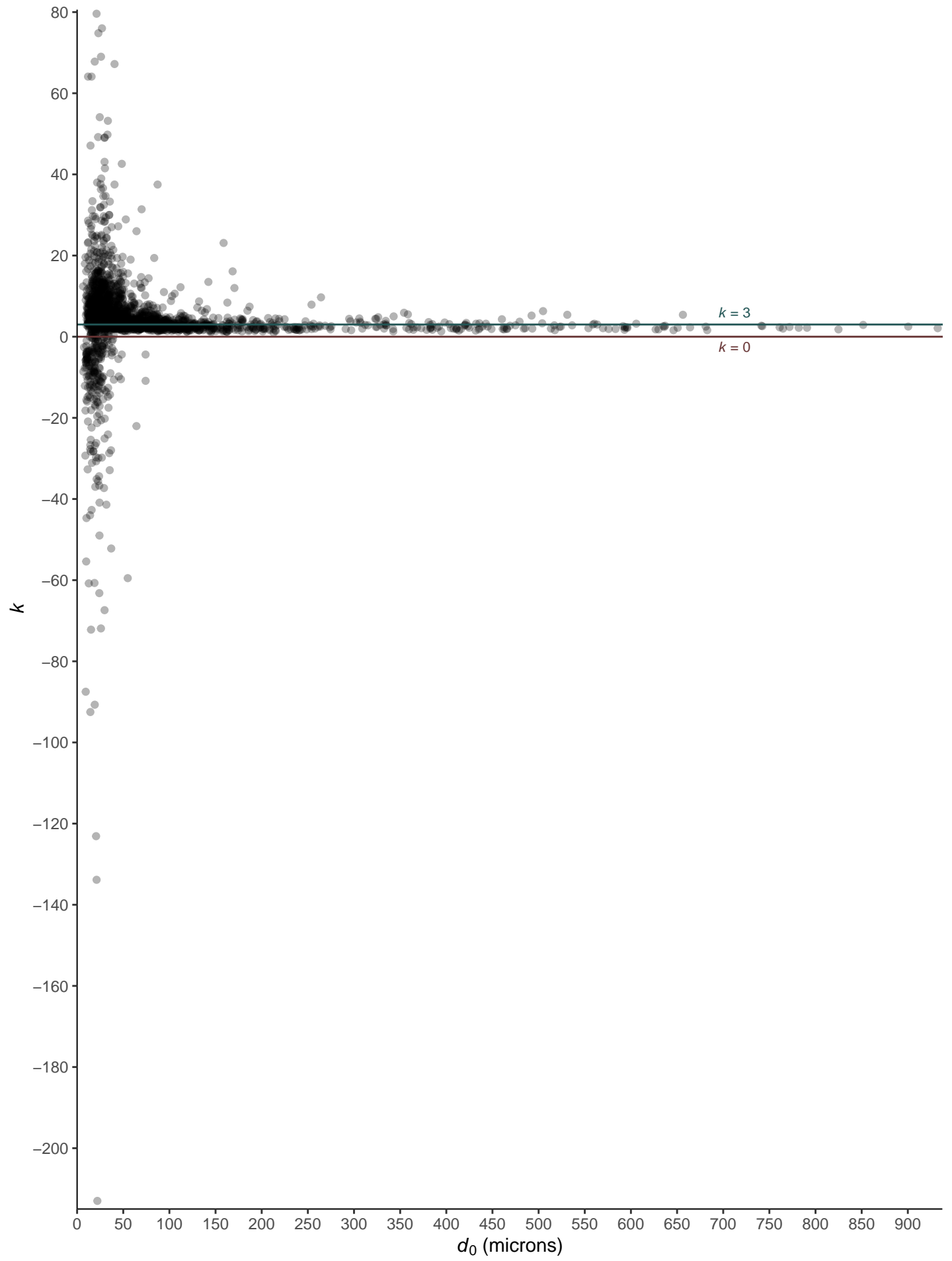

Supplement: Supplementary file 2 — Additional file 2Figure S3. The raw relationship among d0 and k. [file 12915_2021_1130_MOESM2_ESM.pdf]

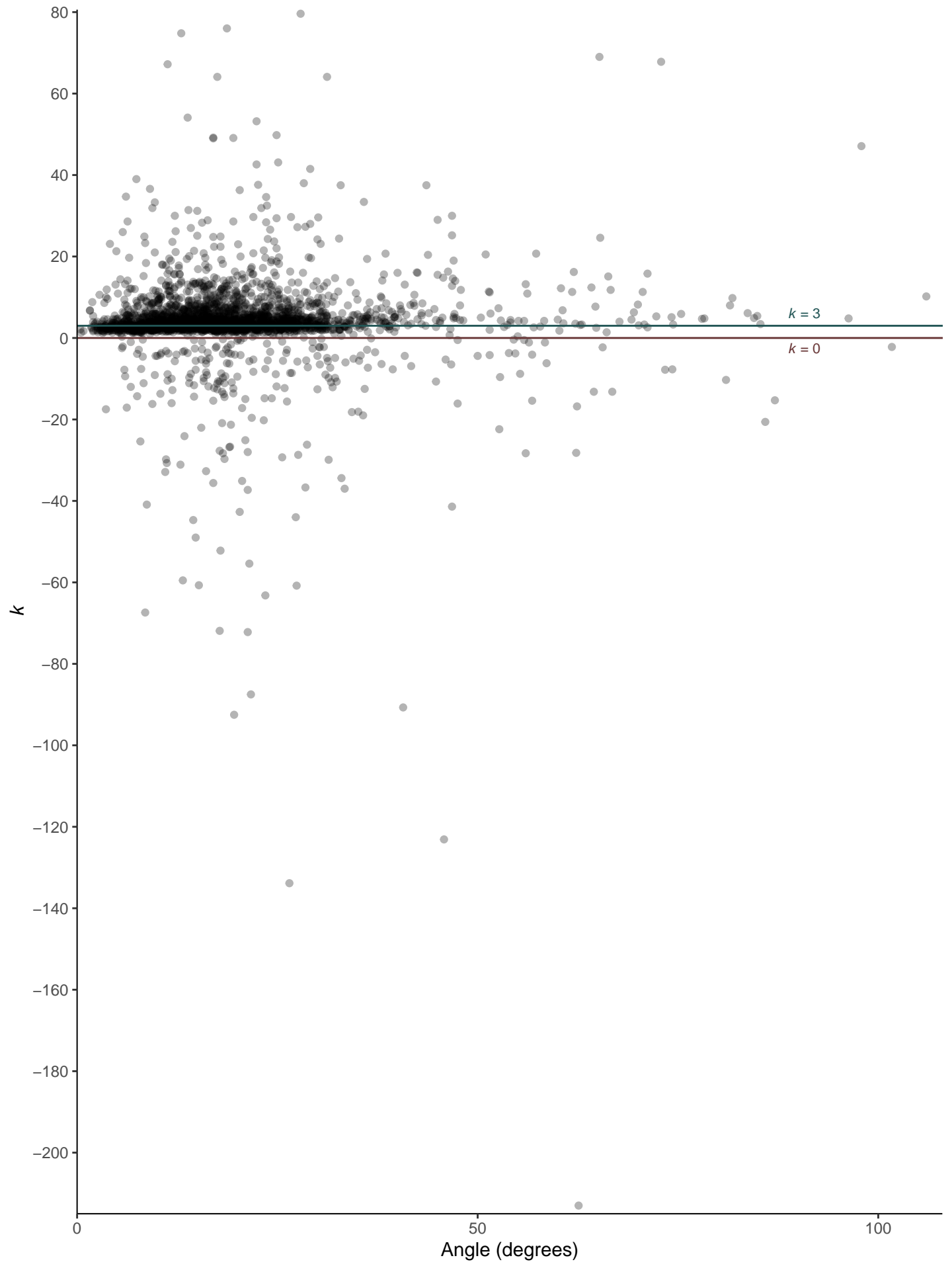

Supplement: Supplementary file 4 — Additional file 4Figure S4. The raw relationship among bifurcation angle and k. [file 12915_2021_1130_MOESM4_ESM.pdf]
