# Supplementary material for: Lepidoptera demonstrate the relevance of Murray’s Law to circulatory systems with tidal flow
Source: BMC Biol. 2021 Sep 15;19:204. doi: 10.1186/s12915-021-01130-0 (PMC8444497; doi:10.1186/s12915-021-01130-0)
Supplement: Supplementary file 3 — Additional file 3 Supplement 2. Supplementary discussion of phylogenetic context. [file 12915_2021_1130_MOESM3_ESM.pdf]

Additional file 3 for: Schachat et al. Lepidoptera demonstrate the relevance of Murray's Law to circulatory systems with tidal flow in *BMC Biology*
Phylogenetic context

As noted in the Methods, we are unable to use evaluate our data with analyses that explicitly account for phylogeny. Our dataset includes many species for which no phylogenetic data are available [1, 2]; the relationships among major superfamilies, such as Gelechioidea and Papilionoidea, or Noctuoidea, Geometroidea, and Bombycoidea, remain uncertain [3, 4, 5, 6]; and the description of new subfamilies and revision of their relationships is ongoing [7, 8], even within families that are relatively well studied [9, 10].

However, even the coarsest view of lepidopteran phylogeny demonstrates that our results are not merely an artifact of phylogenetic autocorrelation. The high variability of k at the low values of d_0 observed in Monotrysia and Gelechioidea, and the conformity to Murray's Law in the wider veins observed in Hepialidae, Papilionoidea, and Bombycoidea, signify that at least one of these shared findings must be the product of convergent evolution rather than a shared ancestral state. Given the dispersion of k among the families and superfamilies of Lepidoptera, there is no possible combination of ancestral states that would preclude convergent evolution of vein diameters—and the corresponding convergence of conformity to Murray's Law. This argument is further strengthened by its applicability to closely related families. Various superfamilies can be divided into families that are stable and monophyletic: Noctuoidea, Pyraloidea, and Papilionoidea all contain at least two families with at least 20 measurements of d_0 in our dataset. Within each of these superfamilies, there is extensive overlap in the values of d_0 in different families (Figure 5). Within Monotrysia, the same argument holds for Adeloidea and non-Coelolepida.

References

[1] Lait LA, Hebert PDN. A Survey of Molecular Diversity and Population Genetic Structure in North American Clearwing Moths (Lepidoptera: Sesiidae) Using Cytochrome c Oxidase I. PLOS ONE. 2018 Aug;13(8):e0202281.

[2] Kaila L. An Annotated Catalogue of Elachistinae of the World (Lepidoptera: Gelechioidea: Elachistidae). Zootaxa. 2019;4632(1):1–231.

[3] Timmermans MJTN, Lees DC, Simonsen TJ. Towards a Mitogenomic Phylogeny of Lepidoptera. Molecular Phylogenetics and Evolution. 2014 Oct;79:169–178.

[4] Heikkilä M, Mutanen M, Wahlberg N, Sihvonen P, Kaila L. Elusive Ditrysian Phylogeny: An Account of Combining Systematized Morphology with Molecular Data (Lepidoptera). BMC Evolutionary Biology. 2015;15(1):260.

[5] Mitter C, Davis DR, Cummings MP. Phylogeny and Evolution of Lepidoptera. Annual Review of Entomology. 2017 Jan;62(1):265–283.

[6] Kawahara AY, Plotkin D, Espeland M, Meusemann K, Toussaint EFA, Donath A, et al. Phylogenomics Reveals the Evolutionary Timing and Pattern of Butterflies and Moths. Proceedings of the National Academy of Sciences. 2019 Oct;p. 201907847.

[7] Wang H, Holloway JD, Wahlberg N, Wang M, Nylin S. Molecular Phylogenetic and Morphological Studies on the Systematic Position of *Heracula Discivitta* Reveal a New Subfamily of Pseudobistonidae (Lepidoptera: Geometroidea). Systematic Entomology. 2019;44(1):211–225.

[8] Park KT, Koo JM, Minet J. Review of the Malagasy Lecithocerid Species Described by Pierre Viette and Deposited in MNHN (Paris), with New Generic Combinations and Descriptions of a New Subfamily and Genus of Momphidae (Lepidoptera: Gelechioidea). Zootaxa. 2020 Sep;4845(2):151–190.

[9] Murillo-Ramos L, Brehm G, Sihvonen P, Hausmann A, Holm S, Reza Ghanavi H, et al. A Comprehensive Molecular Phylogeny of Geometridae (Lepidoptera) with a Focus on Enigmatic Small Subfamilies. PeerJ. 2019;7:e7386.

[10] Zhang J, Cong Q, Shen J, Brockmann E, Grishin NV. Three New Subfamilies of Skipper Butterflies (Lepidoptera, Hesperiidae). ZooKeys. 2019 Jul;861:91–105.
