# Supplementary material for: Lepidoptera demonstrate the relevance of Murray’s Law to circulatory systems with tidal flow
Source: BMC Biol. 2021 Sep 15;19:204. doi: 10.1186/s12915-021-01130-0 (PMC8444497; doi:10.1186/s12915-021-01130-0)
Supplement: Supplementary file 5 — Additional file 5 Supplement 3. Supplementary discussion of generalization of Murray’s Law for insect wings under flow transport, structural function, and total vein network mass constraints. [file 12915_2021_1130_MOESM5_ESM.pdf]

Additional file 5 for: Schachat et al. Lepidoptera demonstrate the relevance of Murray's Law to circulatory systems with tidal flow in *BMC Biology*

Generalization of Murray's Law for insect wings under flow transport, structural function, and total vein network mass constraints

Generalization of Murray's Law

[1] generalized Murray's Law [2] for arbitrary vein networks of arbitrary scale, vein cross-sectional shape and flow profile, assuming the cross-sectional shape is approximately constant in the network and the bifurcation is symmetric. Fluid mechanic assumptions include that the fluid behaves as a continuum i.e. the mean free path length of the molecules should be much smaller than the vein diameter (Knudsen number $\ll 1$, which holds for microfluidic flows [3]), the period over which the unsteady fluid speed varies should be much smaller than the vein diameter divided by the fluid speed (Strouhal number $\ll 1$), and finally, the ratio of the inertial versus viscous forces in the fluid can have any value (Reynolds number is free) [4, 5]. The generalization of Murray's Law results in the same hydraulic radius-cubed relationship between the vein radii before and after the bifurcation as found by Murray for a circular cross section and parabolic flow profile. However, instead of considering the radius of a circular tube, the average hydraulic vein radius is calculated as $r_{hydr} = 2A/P$, with area, A, and the wetted perimeter, P [5]. For a constant cross-sectional shape, the area to perimeter ratio scales the same way as the area-averaged vein radius, $r \stackrel{\text{def}}{=} \sqrt{A/\pi}$, or a representative radius, before and after the bifurcation. This enables us to simplify our radius definition: as long as the vein radius is measured in approximately the same way before and after the bifurcation, it will have to abide to Murray's generalized law in the same fashion as the hydraulic radius.

Continuous structural stiffness law for load carrying veins

In addition to transporting fluid, insect veins also have a structural function. The veins have to support the aerodynamic and inertial forces generated during flight. The corrugated profile of insect wings is a key feature that increases their load carrying ability [6, 7]. Although insect wings must carry load, insects cannot afford their wings to break when overloaded due to impact with the natural environment or turbulent gusts [8]. To avoid structural failure, in particular during hard impact, insect wings have resilin integrated in a number of vein joints [9, 10] that enable them to effectively buckle instead of break when a critical load is reached [11]. Considering this morphological specialization, we assume that vein bifurcations are not optimized for continuous strength, because they fail safely beyond a critical load. Instead we identify the critical role of providing continuous stiffness to bear the (below critical) aerodynamic and inertial loads without deforming too much. This enables insect wings to attain and maintain an effective aerodynamic shape. The vein's cross-sectional shape critically contributes to the bending stiffness of the wing (as well as torsional stiffness

through differential bending). Here we simply consider the vein's contribution to stiffness via the second moment of area in two cases, (1) negligible corrugation and (2) stiffness dominating corrugation. For these two limit cases we determine the relationship between the vein radii before and after the junction for a continuous stiffness contribution along the bifurcating vein.

1. Continuous stiffness along a bifurcating vein in a wing with negligible corrugation. In this case the requirement for a continuous vein stiffness is $I_0 = I_1 + I_2$, in which I is the second moment of area of the vein's cross-sectional shape resisting bending. Given that the second moment of area of a thin walled vein is $I = \pi r^3 t$ [12], we find $\pi r_0^3 t_0 = \pi r_1^3 t_1 + \pi r_2^3 t_2$. So assuming the vein thickness remains approximately constant across the bifurcation, $t_0 = t_1 = t_2$, we find the radii across the bifurcation must relate as in Murray's Law: $r_0^3 = r_1^3 + r_2^3$.

2. Continuous stiffness along a bifurcating vein in a wing with dominant corrugation. This is the case when $h_c/r_v \gg 1$; the stiffness contribution of the corrugation height, h_c, with respect to the neutral line, dominates the contribution from the vein's radius, r_v. In this case the requirement for a continuous vein stiffness is again $I_0 = I_1 + I_2$, in which I is the second moment of area contribution of vein's cross-sectional area located at the extremities of the corrugated wing. The extremities enable the veins to contribute maximal geometric bending stiffness. Given that the second moment of area of a thin walled vein located at the extremity of the corrugation is $I = h_c^2 \cdot 2\pi rt$ [12], we find $h_{c0}^2 2\pi r_0 t_0 = h_{c1}^2 2\pi r_1 t_1 + h_{c2}^2 2\pi r_2 t_2$. Under the reasonable assumptions that the corrugation height and vein thickness remain approximately constant over the bifurcation we find $r_0 = r_1 + r_2$.

To determine which of the two cases best approximates the vein stiffness relationship across the bifurcation, we consider the full second moment of area expression without assuming either one dominates: $I = I_v + I_c = \pi r^3 t + h_c^2 2\pi rt$. The ratio $\xi = I_v/I_c = r^2/2h_c^2$ determines which case applies. For $\xi \ll 1$ the wing's stiffness is corrugation dominated, for $\xi \gg 1$ the second moment of the vein's circular cross section area dominates the wing's stiffness. Given the wing corrugation height is typically several vein radii in highly corrugated paleopteran insect wings [6, 7], it is most parsimonious to assume case 2 represents the ancestral pterygote state for continuous vein stiffness contribution across a bifurcation: $r_0 = r_1 + r_2$. For effectively flat wings (compared to the vein radius) the Murray-like $r_0^3 = r_1^3 + r_2^3$ law applies for continuous stiffness.

In addition to the radius power law, the orientation of the mother vein along the spanwise direction and in particular the bifurcation angle of the daughter vein will modify the continuous stiffness evaluation. The second moment of area of the associated effectively elliptical thin-walled cross sections can be found in [12]. Here we chose to not include these angles in our second moment of area calculation in order to keep our analysis as simple as possible and because there is no obvious correlation between the radius and bifurcation angle (Figure **??**h-n).

The wings of Neoptera are less corrugated than those of Paleoptera [6, 13], and the extent of corrugation can vary within an order [14]. In the few species of Lepidoptera whose wings have been examined in this context, corrugation is minimal—especially among branches of the R vein [15].

References

[1] Stephenson D, Patronis A, Holland DM, Lockerby DA. Generalizing Murray's Law: An Optimization Principle for Fluidic Networks of Arbitrary Shape and Scale. Journal of Applied Physics. 2015 Nov;118(17):174302.

[2] Murray CD. The Physiological Principle of Minimum Work. I. The Vascular System and the Cost of Blood Volume. Proceedings of National Academy of Sciences. 1926;12:207–214.

[3] Rapp BE. Microfluidics: Modeling, Mechanics and Mathematics. Amsterdam: William Andrew; 2016.

[4] Stalder AF, Frydrychowicz A, Russe MF, Korvink JG, Hennig J, Li KC, et al. Analysis of Reynolds, Strouhal and Womersley Numbers in the Healthy Thoracic Aorta. In: Proceedings of the International Society for Magnetic Resonance in Medicine. vol. 19; 2011. .

[5] White FM. Viscous Fluid Flow. MacGraw-Hill, New York. 1991;.

[6] Rees CJC. Form and Function in Corrugated Insect Wings. Nature. 1975;256(5514):200–203.

[7] Jongerius SR, Lentink D. Structural Analysis of a Dragonfly Wing. Experimental Mechanics. 2010;50(9):1323–1334.

[8] Rajabi H, Dirks JH, Gorb SN. Insect Wing Damage: Causes, Consequences and Compensatory Mechanisms. Journal of Experimental Biology. 2020;223(9):jeb215194.

[9] Donoughe S, Crall JD, Merz RA, Combes SA. Resilin in Dragonfly and Damselfly Wings and Its Implications for Wing Flexibility. Journal of Morphology. 2011;272(12):1409–1421.

[10] Rajabi H, Ghoroubi N, Darvizeh A, Dirks JH, Appel E, Gorb SN. A Comparative Study of the Effects of Vein-Joints on the Mechanical Behaviour of Insect Wings: I. Single Joints. Bioinspiration & Biomimetics. 2015 Aug;10(5):056003.

[11] Mountcastle AM, Combes SA. Biomechanical Strategies for Mitigating Collision Damage in Insect Wings: Structural Design versus Embedded Elastic Materials. Journal of Experimental Biology. 2014 Apr;217(7):1108–1115.

[12] Gere JM, Timoshenko SP. Mechanics of Materials. 4th ed. Boston, MA: PWS; 1997.

[13] Engel MS, Davis SR, Prokop J. Insect Wings: The Evolutionary Development of Nature's First Flyers. In: Arthropod Biology and Evolution. Berlin, Heidelberg: Springer Berlin Heidelberg; 2013. p. 269–298.

[14] Breitkreuz LCV, Winterton SL, Engel MS. Wing Tracheation in Chrysopidae and Other Neuropterida (Insecta): A Resolution of the Confusion about Vein Fusion. American Museum Novitates. 2017;(3890):1–44.

[15] Wootton RJ. Leading Edge Section and Asymmetric Twisting in the Wings of Flying Butterflies (Insecta, Papilionoidea). Journal of Experimental Biology. 1993;180(1):105–117.
